# Antioxidative and Anticanceric Activities of Magnolia (*Magnolia denudata*) Flower Petal Extract Fermented by *Pediococcus acidilactici* KCCM 11614

**DOI:** 10.3390/molecules200712154

**Published:** 2015-07-03

**Authors:** Eun-Hye Park, Hyun-Suk Kim, Su Jin Eom, Kee-Tae Kim, Hyun-Dong Paik

**Affiliations:** 1 Department of Food Science and Biotechnology of Animal Resources, Konkuk University, Seoul 143-701, Korea; E-Mails: dms2353@naver.com (E.-H.P.); zmzm852@naver.com (H.-S.K.); insomnia@daum.net (S.J.E.); 2 Bio/Molecular Informatics Center, Konkuk University, Seoul 143-701, Korea; E-Mail: richard44@hanmail.net

**Keywords:** flavonoid, *Pediococcus acidilactici*, *Magnolia denudate*, antioxidative effect, anticanceric activity

## Abstract

In this study, the effects of magnolia (*Magnolia* (*M*.) *denudata*) extract fermentation in increasing the extract’s antioxidative and anticancer activities were investigated. Magnolia was fermented by *Pediococcus acidilactici* KCCM 11614. The total phenolic content was determined by the Folin-Ciocalteu’s method and the antioxidative effects by 1,1-diphenyl-2-picrylhydrazy (DPPH) and ferric reducing ability of plasma (FRAP) assay. Anticancer activity against cancer and normal cells was determined using 3-[4,5-dimethylthiazol-2-yl]-2,5-diphenyltetrazolium bromide (MTT). Total phenolic content during fermentation increased from 38.1 to 47.0 mg gallic acid equivalent (GAE)/g of solid matter. The radical scavenging activity was 91.4% after 72 h fermentation. Fermented magnolia’s antioxidative effect was threefold higher than that of the (non-fermented) control. Fermentation (48 h) increased anticanceric activity against AGS, LoVo, and MCF-7 cancer cells 1.29- to 1.36-fold compared with that of the control, but did not affect MRC-5 (normal) cells, suggesting that fermented magnolia could be used as a natural antioxidative and anticancer agent.

## 1. Introduction

*Magnolia* (*M*.) *denudata* Desrousseaux (Magnoliaceae) is distributed mainly in East and Southeast Asia [1]. Flowering occurs in early spring before leaf flushing and the overall floral display is luxuriant because the individual flowers are large and numerous. *M. denudata* is a pharmacologically safe plant used in different commercially available forms such as teas; infusions; liquids; and capsules for human nutrition. Various biologically active compounds such as eudesmin; magnolin; epimagnoli; neolignans; lignans; phenyl propanoids; sesquiterpenes; and alkaloids have been isolated from *Magnolia* spp. [2]. Other researchers have reported that *M. denudata* is pharmacologically active against disease symptoms such as colds and chronic rhinitis [3]. However; some techniques are necessary to increase their activities for practical application although magnolia flower petals have various bio-functional effects.

Fermentation is used to enhance food quality features such as shelf life, nutritional value, and organoleptic properties [4]. Recently, fermentation has been applied to increase the production and extraction yields of bioactive compounds in the food and pharmaceutical industries [5]. For example, Jo *et al*. [6] reported that ginseng extract fermented by *Aspergillus usamii* had a greater anticanceric activity against HepG2, AGS, and DLD-1 cells than that of the non-fermented extract (NFM). Furthermore, Yoon *et al*. [7] showed that fermentation by *Bacillus subtilis* increased the antioxidant and anticanceric activities of black rice bran.

*Pediococcus* (*P*.) *acidilactici* is a representative lactic acid bacterium used to ferment dairy products [8]. Recently, several researchers have studied the bioconversion process of food components using this strain. Kaur *et al*. [9] for example, reported that fermentation by *P. acidilactici* could convert ferulic acid to vanillin and Michlmayr *et al*. [10] showed that two putative *P. acidilactici* rhamnosidase genes (*ram* and *ram2*) in combination with a bacterial β-glucosidase released the monoterpenes linalool and *cis*-linalool oxide from a wine extract under optimum conditions. 

Oxidative stress is defined as an imbalance in the production of free radicals or reactive oxygen species (ROS) and the antioxidative reactions in the metabolic system of the living cell [11]. ROS, such as the superoxide anion (O_2_^−^), hydrogen peroxide (H_2_O_2_), the hydroxyl radical (OH^•^), and organic peroxides, are produced in cells by the mitochondrial respiratory chain [12]. Ultimately, this imbalance leads to cell damage and that of important biomolecules, potentially compromising cell function [13]. Intracellular proteins and lipids are highly sensitive to oxidative attacks and oxidative modifications of these molecules can increase the risk of cancer [14]. Therefore, the purpose of this study was to ferment *M. denudata* by *P. acidilactici* KCCM 11614 to evaluate the antioxidative and anticancer effects of the fermented extract on various cancer cells for potential application in the food and pharmaceutical industry.

## 2. Results and Discussion

### 2.1. Determination of the Total Polyphenol Content during Fermentation

**Figure 1:**
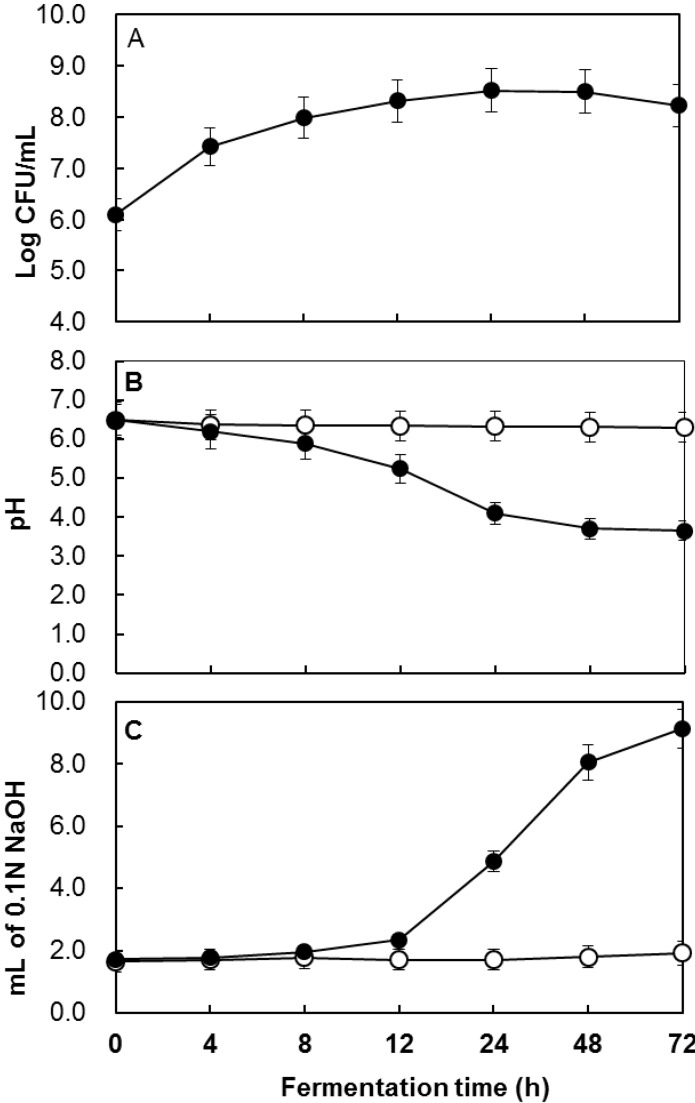
Growth (**A**); pH (**B**), and titrable acidity (**C**) curves during the fermentation of *M*. *denudata* extract by *P. acidilactici* KCCM 11614 at 30 °C for 72 h. ○, Non fermented (control); ●, Fermented.

The stationary phase of the fermentation of the extract with *P. acidilactici* KCCM 11614 was reached after 24 h of culturing at 37 °C. The number of cells increased to 8.5 ± 0.2 colony-forming units (CFU)/mL of culture broth. The pH decreased rapidly from 6.5 to 3.7 ± 0.1, but did not change considerably after 48 h (Figure 1). The total acidity increased from 1.7% to 9.1% ± 0.3% during fermentation. The 72 h fermentation time used in this study was based on these results. 

It is known that the TPC is an important factor in evaluating the biofunctional activities of plant material [15]. As shown (in Table 1), the solid content had decreased from 29.2 ± 0.4 to 23.4 ± 0.2 mg/mL, and the TPC and total flavonoid content had increased from 38.1 ± 0.4 to 47.0 ± 0.1 mg/GAE g of solid and from 27.9 ± 0.1 to 39.6 ± 0.2 mg/quercetin mL, respectively, during the 72 h fermentation. In fermentation of flower petal extract, differences in the polyphenol content can vary according to the resources [5] and microbial strains [16] used for fermentation. Duenas *et al*. [17] reported that microorganisms could hydrolyze complex polyphenols to other simpler and biologically more active compounds. Furthermore, Michlmayr *et al*. [10] reported that *P. acidilactici* produced two distinct glycosyl hydrolase (α-l-rhamnosidases). Therefore, in this study, it was presumed that *P. acidilactici* could effectuate the biotransformation of glycosylated aroma compounds to other components.

**Table 1:** Solid and total phenolic content of *M. denudata* extract fermented by *P. acidilactici* KCCM 11614.

Fermentation Time (h)	Solid Contents (mg/mL)	Total Phenolic Contents (mg GAE ^1^/g of Solid)	Total Flavonoids (mg Quercetin/mL)
0	29.2 ± 0.4 ^2^	38.1 ± 0.4	27.9 ± 0.1
12	26.5 ± 0.2	42.3 ± 0.8	31.3 ± 0.1
24	26.0 ± 0.1	42.4 ± 0.9	33.8 ± 0.2
48	24.5 ± 0.1	48.1 ± 0.5	38.1 ± 0.1
72	23.4 ±0.2	47.0 ± 0.1	39.6 ± 0.2

^1^ Gallic acid equivalent. ^2^ Values are means ± SD of triplicate experiments.

### 2.2. Antioxidative Activity of Fermented Magnolia Extract

To evaluate the antioxidative activity of magnolia fermented by *P. acidilactici* KCCM 11614, several different assays were used to determine the antioxidant activity rather than depending on a single assay, since each method has a different underlying mechanism. Therefore, the methodological limitations and the antioxidative results obtained may be different depending on the analysis method. In this study, the DPPH, β-carotene, and FRAP assay were used of which the results are shown (in Table 2). 

**Table 2:** Antioxidative activities of *M. denudata* extract fermented by *P. acidilactici* KCCM 11614 as determined by the DPPH, β-carotene, and FRAP assay.

Fermentation Time (h)	DPPH Method (%)		β-Carotene Method (%)		FRAP Assay ^1^	
	NFM ^2^	FMPA ^3^	NFM	FMPA	NFM	FMPA
0	80.7 ± 3.1 ^4,d^	85.1 ± 0.3 ^b,c^	84.5 ± 2.4 ^a^	80.7 ± 3.1 ^b^	1.3 ± 0.1 ^a^	1.3 ± 0.1 ^a^
24	82.3 ± 1.4 ^d^	91.5 ± 0.4 ^a^	84.0 ± 1.1 ^a,b^	68.7 ± 1.3 ^c^	1.3 ± 0.1 ^b^	1.2 ± 0.1 ^b^
48	86.0 ± 0.8 ^b^	91.1 ± 1.0 ^a^	84.2 ± 0.5 ^a^	60.4 ± 7.1 ^d^	1.3 ± 0.1 ^a^	1.2 ± 0.1 ^b^
72	83.0 ± 0.8 ^c,d^	91.4 ± 0.3 ^a^	82.6 ± 1.1 ^a,b^	52.5 ± 3.2 ^e^	1.2 ± 0.2 ^b^	1.3 ± 0.2 ^a^

^1^ Unit: mM FeSO_4_ eq. ^2^ NFN, Non-fermented magnolia. ^3^ FMPA, Magnolia fermented by *P. acidilactici* KCCM 11614. ^4^ Values are means ± SD of triplicate experiments. Data were grouped by Duncan’s multiple range test.

DPPH is a stable radical and has been widely used for studying the free radical scavenging activity of several kinds of antioxidants [16]. The DPPH radical scavenging activity of fermented magnolia increased from 85.1% to 91.4% depending on the fermentation time, while those of the NFM (control) were not significantly different (Table 2).

In the β-carotene linoleate bleaching assay, free radical hydroperoxides generated by the auto-oxidation of linoleic acid can bleach β-carotene during incubation at 45–50 °C, which is inhibited by antioxidants because of their hydroperoxide-neutralizing action [18]. Ibrahim *et al*. [19] reported that herbal teas fermented by lactic acid bacteria had significantly greater (*p* < 0.05) antioxidant activity (70%–80%) compared with that of the control (freshly prepared herbal teas). In the present study, 72 h fermented magnolia decreased the β-carotene bleaching activity from 80.7% to 52.5% (Table 2). Hur *et al*. [20] showed that the β-carotene bleaching activity is affected by pH changes. This might have played a role in the current study as the pH decreased from 6.5 to around 3.3 during fermentation.

Using the FRAP method, the antioxidant activity of the fermented and NFM extract was estimated by the extracts’ ability to reduce Fe^3+^-TPTZ to Fe^2+^-TPTZ [21]. The ferric ion-reducing activity of both extract samples was expressed as mM FeSO_4_ eq. At a concentration of 36–25 mg/solid mL, the ferric reducing capacity was not significantly different between the fermented and NFM extract. However, after extraction with 70% ethanol and at a concentration of 0.125–2.0 mg/solid mL to increase the concentration of effective compound in both extracts, the anti-oxidative activity as measured by the FRAP assay correlated with the solid concentration and was increased to 974.8 ± 7.5 µM FeSO_4_ eq at 2.0 mg/solid mL (Table 3).

**Table 3:** Ferric-reducing antioxidative capacity of *Magnolia denudata* extract (70% ethanol) fermented by *P. acidilactici* KCCM 11614.

Conc. (mg/mL)	FRAP Assay ^1^	
	NFM ^2^	FMPA ^3^
0.125	-	20.4 ± 9.4 ^4,h^
0.25	-	118.9 ± 3.0 ^f^
0.5	83.2 ± 1.8 ^g^	226.2 ± 5.7 ^e^
1.0	294.1 ± 1.6 ^d^	537.7 ± 4.1 ^c^
2.0	641.5 ± 6.5 ^b^	974.8 ± 7.5 ^a^

^1^ Unit: µM FeSO_4_ eq. ^2^ NFM, Non-fermented magnolia. ^3^ FMPA, Magnolia fermented by *P. acidilactici* KCCM 11614. ^4^ Values are means ± SD of triplicate experiments. Data were grouped by Duncan’s multiple range test.

The stronger antioxidant activity of lacto-fermented *M. denudata* extract compared with that of the freshly prepared sample (control) was probably due to other factors including, for example, the inherent antioxidant activity of bacteria. Indeed, Yang *et al*. [22] reported that lactic acid bacteria such as *Weissella confusa* and *Lactobacillus plantarum* could have antioxidative effects. It is essential to apply different assays instead of depending on a single assay when determining the antioxidant activity as each method has different underlying mechanisms and methodological limitations. Therefore, the results obtained may be different depending on the analysis method used.

In general, many polyphenols including flavonoids exist as glycosides. Fermentation can cause bioconversion of glycosides. In addition, fermentation can improve antioxidative activity by increasing the release of flavonoids from plant-based foods, which can be a useful method for increasing the supply of natural antioxidative materials. For example, the fermentation-induced structural breakdown of the cell walls of plants may release bioactive compounds and/or induce the synthesis of various other compounds [23,24]. Ng *et al*. [25] also presented that fermentation increase the total phenolic contents in plant and the observed antioxidative activity may be due to the increase in the total phenolic compounds.

### 2.3. In Vitro Determination of Anticanceric Activity

Choi *et al.* [26] reported that *Lactobacillus* strains might constitute anticancer and antioxidative agents due to the presence of soluble polysaccharides in these strains. In the current study, the anticanceric activity of fermented *M. denudata* against four human cancer cell lines (AGS, LoVo, HeLa, and MCF-7) and one human control cell line (MRC-5, normal human lung tissue) was evaluated using the MTT assay. This assay is based on the cleavage of MTT’s tetrazolium ring by the mitochondrial enzyme succinate dehydrogenase, converting MTT to insoluble, purple formazan. Therefore, the number of viable cells can be estimated directly by determining the amount of formazan formed [27]. 

In this study, both NFM and fermented *M. denudata* extract decreased the cell viability of the four different human cancer cell lines used as shown by the MTT assay (Table 4). The anticancer activity of fermented *M. denudata* extract, however, was higher than that of NFM, in particular against AGS, HeLa, and LoVo cells. AGS cells were the most sensitive to both extract treatments. As shown (in Table 4), the anticanceric activity of the 72 h fermented magnolia extract against AGS, LoVo, and MCF-7 cells was 85.0% ± 2.8%, 88.4% ± 1.3%, and 92.0% ± 0.6%, respectively. Moreover, the anticancer effect was 1.29–1.36-fold higher than that of NFM. Interestingly, it seemed that HeLa cells were more resistant to the anticancer effect exerted by the 72 h fermented *M. denudata* extract than any of the other tested cancer cell lines were. On the other hand, the MRC-5 control cells were not significantly affected by fermented *M. denudata* compared with that by NFM (*p* < 0.05). Rutin and quercetin are the main flavonoids in magnolia [28] and are known to have anticancer as well as antioxidative effects [29]. Lignans constitute the main polyphenols found in magnolia [2] and have strong inhibitory effects on cell growth and can induce apoptosis in human cancer cell lines [30]. Hirano *et al*. [31] reported that lignans strongly suppressed the incorporation of [^3^H]thymidine, [^3^H]uridine, and [^3^H]leucine into HL-60 cancer cells. In addition, Simon *et al*. [32] reported that ROS and mitochondria play an important role in apoptosis induction under both physiologic and pathologic conditions. Therefore, the antioxidant activity of fermented magnolia could increase apoptosis of cancer cells. Several researchers have demonstrated that some flavonoids, such as quercetin and luteolin, have apoptotic activity against cancer cells [33,34]. The anti-cancer mechanisms underlying the flavonoids are various, including the induction of cell cycle arrest [35].

Overall, our results suggest that magnolia fermented by *P. acidilactici* can be used in functional foods developed in the food and pharmaceutical industry. However, further studies are needed to both understand the specific anticancer mechanisms underlying the effect of fermented magnolia, and to quantitatively analyze the major effective compounds to be used as potential end-products using high-performance liquid chromatography (HPLC).

**Table 4:** Anticanceric effects of fermented *M. denudata* extract on four different cancer cell lines.

Time ^1^ (h)	Anticancer Activity (%)									
	AGS		HeLa		LoVo		MCF-7		MRC-5 ^2^	
	NFM ^3^	MFPA ^4^	NFM	MFPA	NFM	MFPA	NFM	MFPA	NFM	MFPA
0	61.3 ± 9.9 ^5,c^	76.1 ± 3.2 ^b^	62.4 ± 8.4 ^b^	78.1 ± 3.2 ^a^	72.8 ± 4.1 ^b,c^	73.0 ± 2.7 ^b,c^	64.4 ± 0.9 ^b,c,d^	72.8 ± 1.3 ^d^	82.1 ± 5.1 ^a,b^	83.7 ± 2.1 ^a^
12	59.0 ± 6.3 ^d^	80.8 ± 2.7 ^a,b^	71.9 ± 7.4 ^a,b^	82.6 ± 7.1 ^a^	73.9 ± 5.1 ^b,c^	76.2 ± 9.3 ^b^	66.4 ± 7.1 ^b,c,d^	90.6 ± 2.0 ^b^	81.6 ± 1.1 ^a,b^	82.6 ± 1.1 ^a,b^
24	60.9 ± 4.9 ^d^	82.0 ± 2.9 ^a,b^	76.3 ± 3.9 ^a^	74.7 ± 7.4 ^a^	78.8 ± 5.4 ^b^	88.7 ± 2.1 ^a^	68.7 ± 2.8 ^b,c^	90.7 ± 1.0 ^a^	83.3 ± 2.7 ^a^	84.6 ± 3.0 ^a^
48	57.7 ± 3.5 ^c,d^	83.7 ± 0.3 ^a,b^	70.9 ± 5.8 ^a,b^	75.2 ± 6.2 ^a^	63.8 ± 1.8 ^d^	87.1 ± 0.3 ^a^	62.5 ± 2.6 ^c,d^	91.9 ± 0.2 ^a^	82.4 ± 5.0 ^a,b^	83.6 ± 2.0 ^a^
72	66.0 ± 4.1 ^c,d^	85.0 ± 2.8 ^a^	71.2 ± 3.2 ^a,b^	73.6 ± 6.3 ^a^	66.1 ± 4.0 ^c,d^	88.4 ± 1.3 ^a^	67.5 ± 8.5 ^b,c,d^	92.0 ± 0.6 ^a^	80.4 ± 0.4 ^a,b^	78.0 ± 6.2 ^a,b^

^1^ Fermentation time. ^2^ Normal (control) cell line. ^3^ NFM, Non-fermented magnolia. ^4^ MFPA, Magnolia fermented by *P. acidilactici* KCCM 11614. ^5^ Values are means ± SD of triplicate experiments. Data were grouped by Duncan’s multiple range test.

## 3. Experimental Section 

### 3.1. Strains and Chemicals 

*P. acidilactici* KCCM 11614 was obtained from the Korean Culture Center of Microorganisms (KCCM) in Seoul, Korea. The bacteria were cultured in MRS broth (Difco Laboratories, Detroit, MI, USA) at 37 °C for 12 h and were used to inoculate the extract samples to be fermented. Linoleic acid, β-carotene, 1,1-diphenyl-2-picrylhydrazy (DPPH), 2,4,6-tripyridyl-s-triazine (TPTZ), and 3-[4,5-dimethylthiazol-2-yl]-2,5-diphenyltetrazolium bromide (MTT) were purchased from Sigma-Aldrich Co. (St. Louis, MO, USA).

### 3.2. Cell lines and Culture Conditions

Four cancer cell lines [AGS (human stomach adenocarcinoma), HeLa (human cervical adenocarcinoma), LoVo (human colon adenocarcinoma), and MCF-7 (human breast adenocarcinoma)] and one normal human lung tissue cell line (MRC-5) were purchased from the Korean Cell Line Bank (KCLB; Seoul National University, Seoul, Korea). The AGS, LoVo, and MCF-7 cell lines were incubated in RPMI 1640 medium (Gibco Laboratories, Grand Island, NY, USA) containing 10% heat-inactivated fetal bovine serum (FBS; HyClone, Logan, UT, USA), penicillin (100 U/mL), and streptomycin (100 μg/mL). Both HeLa and MRC-5 cells were cultivated in minimum essential medium (MEM) containing 10% FBS (HyClone), penicillin (100 U/mL), and streptomycin (100 μg/mL). All cells were cultured in a CO_2_ incubator (MCO-18AIC; Sanyo Electric Co., Ltd., Osaka, Japan) at 37 °C under a 5% CO_2_/95% air atmosphere. Adherent cells in the logarithmic growth phase were harvested using 0.25% trypsin (Invitrogen Corp, Carlsbad, CA, USA). Cells were counted with a hemocytometer (Hausser Scientific, Horsham, PA, USA) and were subsequently seeded in new culture dishes and grown to 80% confluence prior to anticanceric activity testing.

### 3.3. Fermentation of Magnolia 

Twenty grams of powdered *M. denudata* were mixed with 2.5 g of peptone (0.5% *w*/*v*) and 10 g of glucose (2% *w*/*v*) in 500 mL of distilled water, and the solution was sterilized at 121 °C for 15 min. After cooling, the mixture was inoculated with 10 mL of the *P. acidilactici* strain (2% *v*/*v*) and cultivated at 30 °C for 72 h. For enumeration of cells, samples were diluted appropriately with 0.1% peptone solution and plated onto MRS agar (Difco Laboratories) media. After culturing, the fermented extract was filtered through a 0.45-μm membrane filter and stored at −20 °C until use. 

### 3.4. Determination of the Total Polyphenol and Flavonoid Content

The total polyphenol content (TPC) in the experimental solution was determined by adding 100 μL of the resultant fermented mixture described under section 2.3 to 2 mL of a 2% aqueous sodium carbonate (Na_2_CO_3_) solution. After a 3-min reaction, 100 μL of 50% Folin-Ciocalteu’s reagent was added to the mixture and the absorbance was measured at 750 nm with a spectrophotometer (2120UV; Optizen, Daejon, Korea) after a subsequent 30-min incubation period. The TPC was determined using a standard curve of gallic acid at 0, 12.5, 25, 50, and 100 μg/mL.

The total flavonoid content of the extract samples was calculated using an aluminum nitrate assay [36]. One hundred microliters of the fermented extract sample, 100 μL of 10% ammonium nitrate (NH_4_NO_3_), 100 μL of 1.0 M potassium acetate (CH_3_COOK), and 4.7 mL of 80% ethanol were mixed. The absorbance of the mixture after incubation at 25 °C for 40 min was measured spectrophotometrically at 415 nm. The total flavonoid content was calculated using the calibration curve of quercetin (Sigma-Aldrich Co.) and the results were expressed as mg of quercetin equivalents per g of solid sample (QE mg/g).

### 3.5. Measurement of the Antioxidative Activity Using the DPPH Method

Two hundred microliters of NFM and fermented extract were added to separate vials containing 1 mL of 100 μM DPPH solution and were shaken. The reaction mixture was allowed to stand for 15 min after which its absorbance was measured at 517 nm. The DPPH free radical scavenging activity of each sample was calculated as follows:

Radical scavenging activity (%) = [1 − (Absorbance_sample_/Absorbance_control_)] × 100 (1)

### 3.6. Measurement of the Antioxidative Activity Using the β-Carotene Bleaching Activity Assay

Twenty milligrams of β-carotene, 44 μL of linoleic acid, and 200 μL of Tween80 were mixed in 10 mL of chloroform. Five milliliters of this mixture were vacuum-dried and re-solubilized with 100 mL distilled water for use as the β-carotene reagent in the assay. To determine the antioxidative activity of each extract sample, 0.5 mL of extract sample was reacted with 4.5 mL of the β-carotene reagent in a water bath at 50 °C. During the incubation, the absorbance at 400 nm was determined at 2 h intervals. The antioxidative activity of the extract samples was calculated as follows:

Antioxidant activity (%) = (Absorbance after reaction/Initial absorbance) × 100
(2)

### 3.7. Determination of Antioxidative Activity Using the Ferric Reducing Ability of Plasma Assay

The ferric reducing ability of plasma (FRAP) assay working reagent was prepared by mixing 300 mM of acetate buffer (pH 3.6), 10 mM of TPTZ solution, and 20 mM of FeCl_3_·6H_2_O in a 10:1:1 ratio immediately before use and by subsequent heating of the resultant mixture to 37 °C [37]. Three hundred millimoles of acetate buffer were prepared by mixing 3.1 g of sodium acetate trihydrate (C_2_H_3_NaO_2_·3H_2_O) with 16 mL of glacial acetic acid, adjusting the total, final volume to 1 L with distilled water. The TPTZ solution was prepared as a solution of 10 mM TPTZ in 40 mM HCl. One hundred microliters of extract sample were mixed with 1.9 mL of the working reagents, the resultant mixture was incubated at 25 °C in the dark for 30 min, and the absorbance was read at 593 nm. A standard curve ranging from 50 μM to 1.5 mM of FeSO_4_ was prepared for the quantitative determination of FeSO_4_ as mM Fe^2+^ and FeSO_4_ equivalents (eq) produced in the samples.

### 3.8. In Vitro Determination of Anticanceric Activity 

The tetrazolium-based colorimetric assay (MTT test) was used for the determination of anticancer activity [38]. One-hundred microliters of the respective cell suspensions were transferred to 96-well microplates. After one day incubation, 100 µL of the extract samples were added to the culture media and the cells were incubated at 37 °C for another 44 h after which the media were discarded and 100 µL of MTT (2.5 mg/mL in phosphate-buffered saline (PBS)) reagent was added to the microplate wells. Following a subsequent incubation for 4 h, the supernatant was discarded and 100 µL of dimethyl sulfoxide (DMSO) was added to each well to dissolve the colored formazan crystals generated by the reaction of the cells with MTT. The absorbance of all samples was measured at 570 nm with a microplate reader (EL311; Bio-Teck Instrument Inc., Seoul, Korea). All experiments were performed in triplicate and the extract samples’ anticanceric activity was calculated as follows:

Anticanceric activity (%) = [1 − (Absorbance_sample_/Absorbance_control_)] × 100 (3)

### 3.9. Statistical Analysis

Each experiment was performed in triplicate. Analysis of variance (ANOVA) was performed using the SPSS software, version 18 (IBM; Chicago, IL, USA). Differences were considered significant at *p <* 0.05.
